# Protecting Science from Abuse Requires a Broader Form of Outreach

**DOI:** 10.1371/journal.pbio.0030218

**Published:** 2005-07-12

**Authors:** Kai M. A Chan, Paul A. T Higgins, Stephen Porder

## Abstract

Students and postdocs at Stanford University have formed an organization dedicated to promoting the use of sound science in policymaking: scienceinpolicy.org.

The politicization of science may be as old as science itself. Famously, Galileo's championing of the theory that the Earth revolves around the sun met with staunch political opposition as a perceived challenge to the authority of the Catholic church. In the Soviet Union, Lysenko rejected the widely held chromosomal theory of inheritance in favor of a theory of environmental influences that aligned more closely with the philosophical underpinnings of communism. More recently, the assertion of the South African president Thabo Mbeki that AIDS is not caused by HIV flew in the face of decades of research and threatened to undermine proper treatment of the disease. In the view of many, science under the current United States administration is also under threat. Underlying many government policies that depend upon or affect science is a pattern by which evidence—which decision makers could use to craft well informed policies—is changed into a subjective tool for political or ideological goals [1–8]. The resulting perceptions of government hostility toward science threaten to drive frustrated federal scientists from agencies and to undermine the already flagging public respect for science. Ultimately, blame for the tension between science and politics does not lie with politicians alone. The minor role that scientists have played in the public arena allows such tensions to persist, and to grow elsewhere.

To confront this issue, we joined with other graduate students and postdocs at Stanford University to initiate scienceinpolicy.org—a grassroots organization dedicated to promoting the use of sound science in US policymaking. Assessing the major environmental issues in the public eye, we found and categorized a widespread pattern of manipulation and suppression of environmental science affecting issues as diverse as climate change, forestry policy, endangered species protection, clean water, and air pollution on our website (www.scienceinpolicy.org). For example, when the US Environmental Protection Agency sought to warn the public of health threats from air pollution following September 11, 2001, the White House edited press releases to significantly change the meaning from one of warning to placation. Because we researched and cited both news reports and the primary literature, and distributed our analysis for friendly peer review, our analysis attained a level of scientific credibility beyond that of regular media. [Fig pbio-0030218-g001]

**Figure pbio-0030218-g001:**
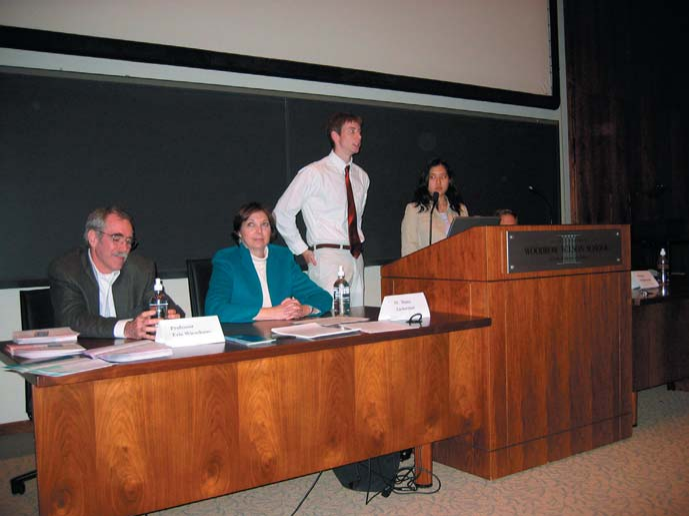
Professor Eric Weischaus (Princeton Professor of Molecular Biology, Nobel laureate), Dr. Diana Zuckerman (President of the National Research Council for Women and Families) and two student organizers participate in a public forum at Princeton University on scientific integrity in policymaking (Photo: Princeton Environmental Action)

We alerted our colleagues to the information we had gathered by circulating emails, thereby 1) receiving additional informal peer review to ensure that our assessment and criticisms were fair and accurate, 2) empowering ourselves for additional outreach efforts such as writing op-eds and letters to the editor, appearing on radio programs, etc., and 3) establishing connections with other groups working to protect science. For example, when the Union of Concerned Scientists (Cambridge, Massachusetts, United States of America) spearheaded a more professional campaign with Nobel laureates and other prominent scientists, we seized the opportunity to act as a grassroots arm of their effort. We proposed a series of public forums on scientific integrity in policymaking at university campuses across the country, and initiated and promoted several of these events through our distribution list of almost 2,000 environmental scientists. At Princeton University (Princeton, New Jersey, United States of America), hundreds of students and community members packed the auditorium to overflowing.

Despite the favorable response, it was a challenge to marshal sufficient time and effort to execute an effective campaign. Impeding our progress was the very structure and culture of the academic community, in which research is valued above all else, and energy expended towards any other end is energy that could have been spent attempting to advance one's career in an extremely competitive job market. Despite apparently universal agreement on the need for scientist outreach to benefit both society and science (e.g., [9,10]), outreach is not encouraged institutionally, and may be actively deterred [11]. Outreach only rarely benefits young scientists striving for jobs and promotions. If adamant researchers persist with civic engagement, they frequently find themselves unprepared by a graduate training that emphasizes research skills almost exclusively.

Calling for greater scientist engagement in society is appropriate but cannot succeed without the institutional changes necessary to promote such behavior. Such changes will only occur when scientists change their own institutions. Reward structures, hiring and promotion decisions, and the structure and content of graduate training are all amenable to change by motivated students, postdocs, professors, and institutional officials. Since a healthy relationship between science and society may depend upon a marked reform of scientific training and oversight, scienceinpolicy.org is expanding its focus to encourage institutional changes that promote researchers reaching out to the media, the public, and policy makers.

To advance our vision of a vibrant academy that makes important contributions to a thriving society, we advocate a variety of concrete changes to academic culture and institutions. Graduate courses on effective outreach and communication with the media would empower interested students with needed abilities. New fellowship programs and awards for engagement like the Aldo Leopold Leadership Program (Stanford, California, United States of America) would provide training, camaraderie, and motivation for communicating with policy makers and the public. Institutional acceptance and/or support for an explicit time tithe for outreach would assuage the feeling that engaging with society means neglecting academic duty. New funding for outreach to, and partnerships with, governmental institutions, NGOs, etc, would provide both enticement and capital. Explicit consideration of outreach in promotion decisions—as exists in at least one institution—would reduce the opportunity costs associated with outreach and affirm the value of these activities [12]. We need to discuss and evaluate the numerous ways to appropriately encourage civic engagement, starting now.

One need only to look to the broader public for evidence that science is facing a crisis in the US. The public continues to debate the teaching of evolution in public schools. Meanwhile, the popularity of Michael Crichton's novel *State of Fear*—in which scientists are complicit in environmentalists' plot to manipulate the public by vastly exaggerating the threats of climate change—promotes and amplifies the already debunked claims of climate-change naysayers. But such public responses are only symptoms of a broader disconnect between science and society, a result of our academic isolation in the Ivory Tower.

## References

[pbio-0030218-b1] Union of Concerned Scientists (2004). Scientific integrity in policy making: Further investigation of the Bush administration's misuse of science.

[pbio-0030218-b2] Union of Concerned Scientists (2004). Scientific integrity in policy making: An investigation into the Bush administration's misuse of science.

[pbio-0030218-b3] United States House of Representatives Committee on Government Reform—Minority Staff (2003). Politics and science in the Bush administration.

[pbio-0030218-b4] Waxman HA (2005). Politics and science: Investigating the state of science under the Bush administration. http://democrats.reform.house.gov/features/politics_and_science/index.htm.

[pbio-0030218-b5] Chan KMA, Porder S, Higgins PAT, Kramer SB (2004). Concern is more than just ‘ruffled feathers’: If a government abuses science to justify its policies, scientists have a duty to speak out. Nature.

[pbio-0030218-b6] Porder S, Chan KMA, Higgins PAT (2004). Scientists must conquer reluctance to speak out: When science is under political assault, keeping a dignified silence is counterproductive. Nature.

[pbio-0030218-b7] Higgins PAT (2004). The Bush administration and climate change. Science.

[pbio-0030218-b8] Association of Reproductive Health Professionals (2005). Preserving core values in science.

[pbio-0030218-b9] Marincola E (2003). Research advocacy: Why every scientist should participate. PLoS Biol.

[pbio-0030218-b10] Reid WV (2004). Bridging the science–policy divide. PLoS Biol.

[pbio-0030218-b11] Diamond JM (2004). Lessons from environmental collapses of past societies.

[pbio-0030218-b12] Schlesinger WH (2003). Scientists must boldly address important environmental issues. The Herald Sun (Durham).

